# Supplementary material for: Standardized Computer-Assisted Analysis of PRAME Immunoreactivity in Dysplastic Nevi and Superficial Spreading Melanomas
Source: Int J Mol Sci. 2023 Mar 28;24(7):6388. doi: 10.3390/ijms24076388 (PMC10094429; doi:10.3390/ijms24076388)
Supplement: Supplementary file 1 [file ijms-24-06388-s001.zip › ijms-2256714-supplementary.pdf]

Supplementary Table 1: Receiver operating characteristic curve (ROC-curve) and the area under the curve (AUC) values of the entire lesion and the epidermis (level 1=increased intensity threshold).

Area under the ROC Curve					
				95% confidence interval	
Cells/mm²	AUC	Std. Error	Asymptotic significance	Lower bound	Upper bound
Entire lesion - baseline	0.866	0.044	p<0.001	0.780	0.953
Entire lesion - level 1	0.873	0.043	p<0.001	0.789	0.957
Epidermis - baseline	0.901	0.037	p<0.001	0.829	0.973
Epidermis - level 1	0.900	0.037	p<0.001	0.827	0.973

Supplementary Table 2: Coordinates of the ROC Curve of the entire lesion (cells/mm²).

Positive if greater than or equal To[a]	Sensitivity	1 - Specificity
-1.0000	1.000	1.000
0.4874	1.000	0.844
1.9404	1.000	0.813
2.9725	1.000	0.781
3.042	1.000	0.750
3.136	1.000	0.719
3.351	1.000	0.688
3.971	1.000	0.656
4.6355	0.969	0.656
4.8645	0.969	0.625
6.83	0.969	0.594
9.5525	0.969	0.563
13.36	0.969	0.531
17.245	0.938	0.531
18.505	0.906	0.531
19.455	0.906	0.500
23.83	0.906	0.469
29.27	0.906	0.438
31.72	0.906	0.406
33.88	0.906	0.375
36.685	0.875	0.375
42.04	0.875	0.344
47.05	0.875	0.313
48.94	0.844	0.313
52.55	0.813	0.313
58.255	0.781	0.313
62.15	0.781	0.281
63.29	0.781	0.250
75.495	0.781	0.219
88.85	0.750	0.219
90.135	0.750	0.188
90.35	0.719	0.188
92.295	0.719	0.156
97.67	0.719	0.125
105.995	0.688	0.125
114.86	0.656	0.125

120.52	0.625	0.125
124.935	0.625	0.094
148.46	0.625	0.063
180.23	0.594	0.063
203.66	0.563	0.063
236.57	0.531	0.063
299.245	0.531	0.031
342.985	0.500	0.031
372.255	0.469	0.031
447.045	0.438	0.031
507.405	0.406	0.031
576.32	0.375	0.031
638.465	0.344	0.031
645.31	0.313	0.031
660.68	0.281	0.031
691.555	0.250	0.031
711.555	0.250	0.000
783.47	0.219	0.000
909.115	0.188	0.000
1011.47	0.156	0.000
1075.75	0.125	0.000
1101.85	0.094	0.000
1219.55	0.063	0.000
1382.9	0.031	0.000
1436	0.000	0.000
